# A Noncontact Dibutyl Phthalate Sensor Based on a Wireless-Electrodeless QCM-D Modified with Nano-Structured Nickel Hydroxide

**DOI:** 10.3390/s17071681

**Published:** 2017-07-21

**Authors:** Daqi Chen, Xiyang Sun, Kaihuan Zhang, Guokang Fan, You Wang, Guang Li, Ruifen Hu

**Affiliations:** 1 State Key Laboratory of Industrial Control Technology, Institute of Cyber Systems and Control, Zhejiang University, Hangzhou 310027, China; 11332013@zju.edu.cn (D.C.); 21532085@zju.edu.cn (X.S.); zhangkaihuan@zju.edu.cn (K.Z.); king_wy@zju.edu.cn (Y.W.); guangli@zju.edu.cn (G.L.); 2 School of Chemistry and Chemical Engineering, Yangzhou University, Yangzhou 225002, China; gkfan@yzu.edu.cn

**Keywords:** wireless-electrodeless QCM with dissipation, dibutyl phthalate, overtone

## Abstract

Dibutyl phthalate (DBP) is a widely used plasticizer which has been found to be a reproductive and developmental toxicant and ubiquitously existing in the air. A highly sensitive method for DBP monitoring in the environment is urgently needed. A DBP sensor based on a homemade wireless-electrodeless quartz crystal microbalance with dissipation (QCM-D) coated with nano-structured nickel hydroxide is presented. With the noncontact configuration, the sensing system could work at a higher resonance frequency (the 3rd overtone) and the response of the system was even more stable compared with a conventional quartz crystal microbalance (QCM). The sensor achieved a sensitivity of 7.3 Hz/ppb to DBP in a concentration range of 0.4–40 ppb and an ultra-low detection limit of 0.4 ppb of DBP has also been achieved.

## 1. Introduction

Dibutyl phthalate (DBP) is a kind of phthalate plasticizers which has been used as an additive in lots of applications, including nail lacquers, food packaging films, adhesives, pharmaceuticals and even toys for children [1,2,3] in the last few decades. As a result, DBP vapor has become ubiquitous in the environment. However, being exposed to DBP vapor can be harmful to human health. Recent research has found that DBP is a reproductive and developmental toxin to males and DBP has been considered as an endocrine disrupting compound (EDC) [4,5]. As air is an important medium for DBP to enter human bodies, a lot of research has assessed the concentration of DBP in indoor air (1269–7104 ng/m^3^), indoor dust (4.4–2300 mg/kg) and ambient air (PM_2.5_ associated 8.72 μg/m^3^, PM_10_-associated 12.90 μg/m^3^) [6,7,8]. In the Proposition 65, the maximum allowable dose levels (MADL) for DBP is 8.7 μg/day [9], which means if breathing contains 10% of the DBP intake, the concentration of the DBP vapor in the air should be lower than 0.087 μg/m^3^ (6.8 ppb) to ensure safety.

Considering the facts above, the need for a DBP sensor that can monitor the concentration of DBP vapor in air is very urgent to avoid adverse effects, especially for people who work in a plastic production workshop. Such people could easily suffer from staying for a long time in an environment that contains relatively high concentrations of DBP vapor. The common methods used to determine the DBP concentration in air are gas chromatography (GC) and gas chromatography-mass spectrometry (GC-MS). Both the methods can measure extremely low concentration (respectively 1218–2453 ng/m^3^ and 52–1100 ng /m^3^) [10,11], but they both take considerable time and are expensive, which disagrees with the demand for fast and cheap monitoring in daily life.

For the fast and cheap detection of DBP in air, the quartz crystal microbalance (QCM) sensor is a popular choice because of their high sensitivity and room temperature working conditions. Different sensing material have been developed to modify the QCM sensor in order to monitor DBP in the air. Wang [12] deposited the polyaniline nanofibers on the electrode of a quartz crystal oscillator as the sensitive film and detected the DBP in the range of 20–1000 ppb with a detection limit of 20 ppb. Hu and Zhang [13,14] respectively used nano-structured nickel hydroxide and Au-decorated ZnO to coat QCM sensors and achieved a detection range of 5–40 ppb and 2–30 ppb with detection limits of 5 ppb and 2 ppb.

However, conventional QCM sensors have some drawbacks which limit their application: (1) electrodes on both sides of the quartz disc deteriorates the sensitivity of the sensor and limits the available frequency because they are also mass adsorbed on the oscillator’s surface [15]; (2) some coating materials for the oscillators were restricted. For example, bioactive materials were hard to coat on the QCMs for a long time because of the biotoxicity of the metal electrodes; (3) this configuration is only applicable in the case when the sensing film is firmly attached to the quartz crystal surface, oscillating rigidly together with the crystal throughout the experiment [16]. When there are visco-elastic changes of the deposited film, the mass would not be a linear relationship with the mass loaded.

To solve all the problems mentioned above, we developed a wireless-electrodeless QCM with a dissipation system. This system could simultaneously monitor the frequency and the dissipation value in a noncontact way, which means that it combines both the advantages of the quartz crystal microbalance with dissipation (QCM-D) system [17] and the wireless-electrodeless QCM system [18,19,20,21,22,23,24]. Based on this system, a DBP sensor was constructed and nano-structured nickel hydroxide [14] was deposited as the sensing film. The depositing load of the sensing film was optimized and the sensor performance including sensitivity, selectivity and limit of detection was evaluated. Then the operating frequency was raised to 3rd harmonics, and the same experiment was carried out. With the homemade sensor applied in DBP detection, drilling in the chamber for wires was avoided, and thus reduced the risk of leaking of the test vapor. Meanwhile, the raised operating frequency improved the sensitivity of the sensor (7.3 Hz/ppb to DBP in a concentration range of 0.4–40 ppb) and achieved lower limit of detection (0.4 ppb). Moreover, the signal-to-noise ratio (SNR) of the signal was improved and the system was thus more stable. Although influence on performance of the sensor by fluctuation of temperature were discussed in some research [25,26,27], we did not discuss it in this thesis because the temperature in production workshops is quite stable in order to ensure the stability of product quality. In conclusion, the wireless-electrodeless QCM-D system is a good and new choice to achieve fast and highly sensitive detection of volatile organic compounds (VOCs).

## 2. Experimental Section

### 2.1. Materials

All the chemicals and reagents were analytical grade. Nickel dichloride, ammonia, dibutyl phthalate (DBP), diethyl phthalate (DEP), dimethyl phthalate (DMP), ethanol, chloroform, ethyl acetate, acetic acid, acetaldehyde (40% *v*/*v*) and benzene were purchased from Sigma–Aldrich (Shanghai, China). The AT–cut 6.0 MHz quartz crystal discs were purchased from Kesheng Electronics (Yantai) Ltd., Yantai, China.

### 2.2. The Wireless-Electrodeless QCM-D System

**Figure 1:**
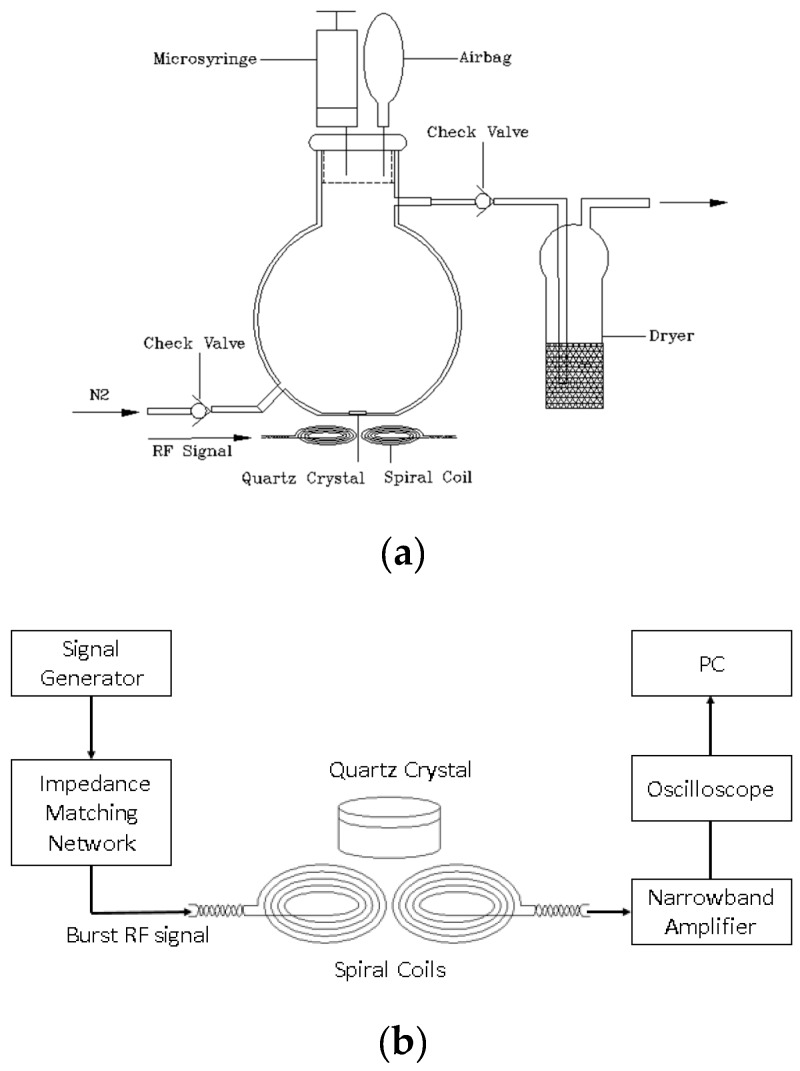
(**a**) The homebuilt electrodeless quartz crystal microbalance (QCM) gas chamber. Two spiral coils are placed outside the chamber and right below the quartz plate. They are used to radiate the electric field, which excites the quartz oscillator and receives the vibrational signals of the quartz oscillator. The quartz oscillator is placed at the bottom of the chamber and above the middle of two coils. (**b**) The whole structure of the wireless-electrodeless quartz crystal microbalance with dissipation (QCM-D) system. The signal generator generates the burst radio frequency signal and finally sends it to the transmitting coil. The other coil receives the signal and then sends it to the narrowband amplifier then to the oscilloscope and finally to the PC for analyzing.

Base on the traditional QCM-D system and the wireless-electrodeless QCM systems, a homemade QCM-D system was built combining their advantages. Several instruments was used to form this system, the function generator was Tektronix AFG3102C (Tektronix, Beaverton, OR, USA), the oscilloscope was Tektronix TDS5054B (Tektronix, Beaverton, OR, USA), the narrow band amplifier was homemade and the impedance matching network was a HF automatic antenna tuner (mAT-125E). Figure 1a shows the homebuilt electrodeless QCM-D gas chamber. The AT-cut 6.0 MHz quartz plate was placed at the bottom of the chamber where a 9 mm diameter blind hole was machined to fix the location of the quartz plate. Two spiral coils were placed below the chamber and right below the quartz plate. The distance between the coils and the quartz plate was 3 mm. The volume capacity of the chamber was 1 L. At the top of the chamber, a small plastic airbag was used to keep the pressure as 1 atm.

In order to make the whole system to work, circuits and signal processing units were needed to form a whole system. In Figure 1b, to excite and detect the vibrations of the quartz oscillator, a radio frequency (RF) signal whose frequency was very close to the resonant frequency of the quartz crystal was generated by the function generator and sent to the transmitting coil. After that, an alternating electric field was generated and the quartz oscillator was excited because of the converse piezoelectric effect. After the quartz plate was steadily vibrating, the RF signal was removed and the receiving coil received the exponentially decaying mechanical vibration signals of the quartz plate through the piezoelectric effect. The received signals entered a narrowband amplifier then to the oscilloscope so that the damping vibration signal was recorded. Then, the PC was used to real-time analyze the frequency and dissipation value. During the experiment, the resonant frequency of the quartz oscillator will change, but we did not change the frequency of the RF signal after the resonant frequency of the quartz crystal reached the base line in case of an effect on the vibration state of the quartz oscillator.

### 2.3. Fabrication of a QCM Gas Sensor

The preparation of nano-Ni(OH)_2_ was carried out in the same way as the previous work [14]. Before the experiment, the quartz plates should be coated with nano-Ni(OH)_2_ as the sensing film. The AT–cut 6.0 MHz quartz plates were washed with deionized water and anhydrous alcohol for 15 min respectively. Then they were dried by high–purity N_2_ at room temperature. Then 10 mg nano-Ni(OH)_2_ was added to 1 mL deionized water in a burette and dispersed by ultrasonication to form a 10 mg/mL Ni(OH)_2_ solution. Finally, the nano-Ni(OH)_2_ solution sample was evenly smeared onto the surface of the quartz plates and the quartz plates were dried for 24 h at room temperature. The QCM sensors coated with nano-structured Ni(OH)_2_ sensing film were obtained.

### 2.4. Preparation of Measured Vapors

The vapors to be measured in the experiment were DBP, DEP, DMP, ethanol, chloroform, ethyl acetate, acetic acid, acetaldehyde (40% *v*/*v*) and benzene. All of them are liquid state at room temperature. To form a mixed gas with a known concentration, small amounts of the analyte solutions were injected into 2 L airbag full of high–purity N_2_. According to the Equation (1), the volume of every kind of the analyte solutions injected were calculated except for DBP, DEP and DMP.

(1) $$V_{x}\;=\;\frac{V\times C\times M}{22.4\times d\times P}\;\times \;10^{-9}\;\times \;\frac{273+T_{R}}{273+T_{B}}$$

where *V_x_* is the analyte solution (mL), *V* is the volume of the airbags (mL), *C* is the target concentration of the measured vapors (ppm), *M* is the molecular weight of the analyte, *d* is the density of the analyte solution (g/cm^3^), *P* is the purity of the analyte solution (%), *T_R_* is the room temperature and *T_B_* is the airbag temperature. Through this method, vapors whose concentration were 1 ppm of ethanol, chloroform, ethyl acetate, acetic acid, acetaldehyde and benzene were obtained.

Last, in order to form a known concentration mixed gas of DBP, DEP and DMP, an excess amount of them was respectively injected into the 2 L airbag to get saturated vapors. The concentration of the saturated vapors were 0.84 ppm (DBP), 2.76 ppm (DEP) and 4.05 ppm (DMP) at 25 °C.

### 2.5. Gas Sensing Experiments

The quartz plate with sensing film was first put in the blind hole of the chamber. Before the experiment, the frequency of the burst RF signal was set to a suitable value to guarantee the oscillation of the quartz plate. Then, high-purity N_2_ was continuously injected to purge the chamber and to desorb the sensors at the velocity of 120 L/min until the resonant frequency of the sensor reached a stable baseline. After this, the frequency of the burst RF signal was tuned to approach the baseline frequency and held. In every experiment, N_2_ was first injected to desorb the sensor until its resonant frequency returned to the baseline. Then precise volumes of mixed gas of the analyte were injected into the chamber and left to stand for 5 min to obtain appropriate concentrations of the vapors in the chamber. At the same time, the frequency was continuously monitored and the frequency differences were the responses. To maintain the balanced pressure in the gas chamber when injecting the gas samples, a small elastic bag was connected to the chamber through a thin catheter (see Figure 1a).

## 3. Results and Discussion

### 3.1. SEM Morphology

**Figure 2:**
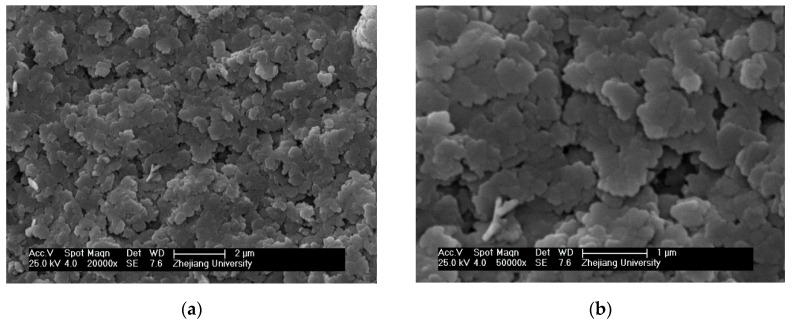
SEM morphology of the nano–Ni(OH)_2_ sample at different scales. (**a**) ×20,000, (**b**) ×50,000.

The scanning electron microscopy (SEM) technique was used to investigate the morphology and nanostructure of the Ni(OH)_2_ film. In Figure 2a,b, the Ni(OH)_2_ sample presented a sheet-like morphology.

### 3.2. Optimization of the Thickness of the Sensing FILM

**Figure 3:**
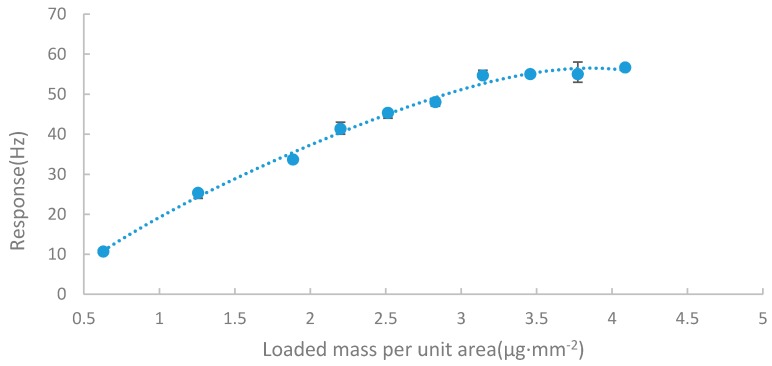
Response curve of the nano-Ni(OH)_2_ QCM sensors to 24 ppb dibutyl phthalate (DBP) with different loaded mass of sensing material.

The thickness of the sensing film on the quartz plate was a key factor to the sensitivity. Thicker sensing film could provide more adsorption sites and thus more analyte molecules would be adsorbed in the same concentration. However, when there were too many layers of the sensing material on the film, the analyte molecules could not attach to the inner sites and the adsorb mass would reach saturation. Worse, if the sensing film was too thick, it would deteriorate the sensing ability of the QCM sensors because the sensing film is also the adsorb mass of the QCM. Based on this, 10 sensors with different thickness sensing films were made by controlling the volume of the nano-Ni(OH)_2_ solution coated on the QCM to optimize the film thickness. The responses of all these sensors to 24 ppb DBP vapor were measured and are shown in Figure 3. The response of the sensor is proportional to the thickness of the nano-Ni(OH)_2_ film when the loaded mass is below 3.14 μg/mm^2^, and then reaches a saturation level after that. In order to achieve a fast response and avoid error caused by the coating process, the optimal thickness of nano-Ni(OH)_2_ film was chosen as 3.46 μg/mm^2^, a little thicker than the minimum stable point.

### 3.3. Selectivity

**Figure 4:**
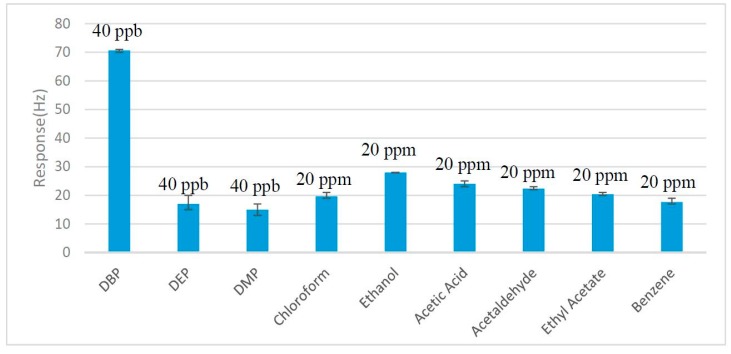
Responses of the nano-Ni(OH)_2_ QCM sensor to various organic vapors. The concentration of DBP, diethyl phthalate (DEP) and dimethyl phthalate (DMP) was 40 ppb, while other vapors were 20 ppm. The response of DBP was far bigger than the inferences.

To investigate the selectivity of the sensing film, diverse VOCs interferences, including two other plasticizer vapors, DEP and DMP, and several conventional solvents like ethanol, chloroform, ethyl acetate, acetic acid, acetaldehyde and benzene, were tested. Among them, DEP and DMP were tested at 40 ppb and the other conventional solvents were tested at 20 ppm. Figure 4 indicates that the response of the nano-Ni(OH)_2_ QCM sensor to the DBP vapor is greater than that of the other two plasticizer vapors under the same concentration. Moreover, although other conventional inferences were 500 times the concentration of DBP vapor, the responses were still far smaller than that of DBP. These results indicated that the affinity of the sensor to plasticizer vapors were much better than other conventional inferences and suggested that the sensor can detect plasticizer vapors at ppb levels. The reason was possibly that DBP, DEP and DMP contain more hydrogen bond acceptors (4) than the other interferences (≤2), which means they are more easily able to interact with the −OH groups on the surface of the nano-structured nano-Ni(OH)_2_ through hydrogen bonding so that the physical adsorption was facilitated. Additionally, the benzene ring and the symmetrical molecular structure of DBP, DEP and DMP lead to their good hydrophobicity, which contributes to their affinity. Finally, among DBP, DEP and DMP, the response amplitudes decreased in turn, the length of the alkyl chains in the target analytes may account for this [13].

### 3.4. Sensitivity at the Fundamental Frequency

**Figure 5:**
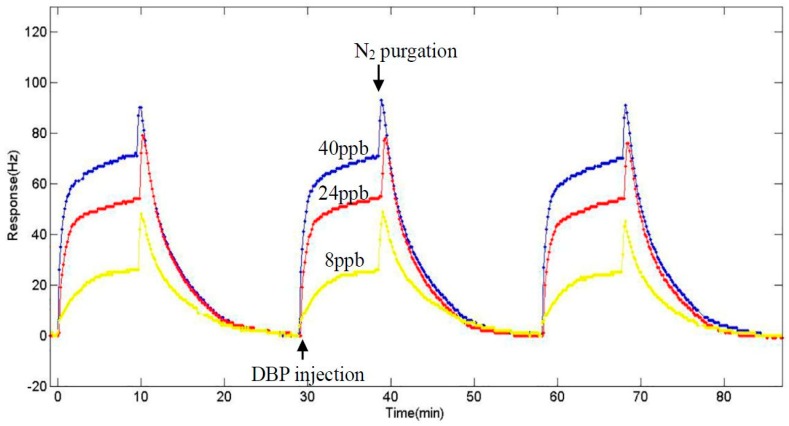
Response of the nano-Ni(OH)_2_-coated QCM sensor to 8, 24, and 40 ppb (from bottom to top) of DBP.

**Figure 6:**
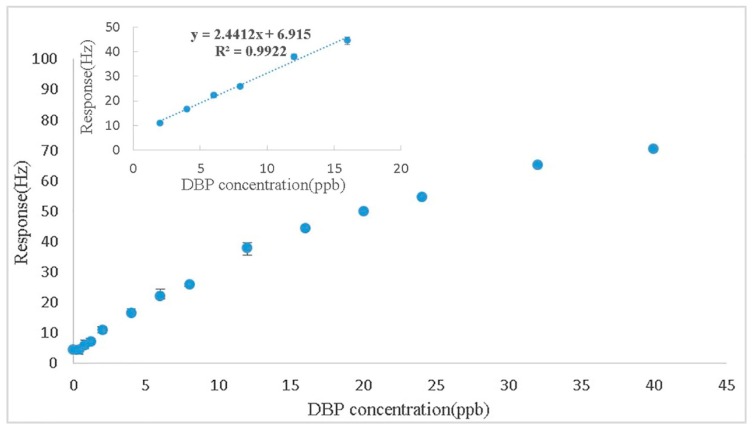
Calibration curve of the nano-Ni(OH)_2_-coated QCM sensor to different concentrations of DBP vapor. The inset indicates the calibration curve of the linear range.

After optimizing the thickness of the sensing film and testing the selectivity, the QCM sensor with the film thickness of 3.46 μg/mm^2^ was tested in different concentrations of DBP vapor that ranged from 0.4 ppb to 40 ppb. For each concentration, three cycles were carried out to test the repeatability and the stability of the sensor. Figure 5 shows the dynamic response curves of the QCM sensor when three different concentration DBP vapor were injected into the chamber. From the figure we can know that the response time is less than 300 s when the concentration is higher than 8 ppb and the recovery time is nearly 600 s for all concentration, which can satisfy the fast monitor of the concentration of the DBP vapor. The response time was longer when the concentration was lower, probably because of smaller concentration gradient difference that slow down the absorption process. Moreover, the response curves were extremely stable with the error below 1 Hz, showing good stability and accuracy of the new system. The noncontact configuration rather than using a long wire to connect the oscillator to the circuit was the key reason. Thanks to this, good SNR (>50) of the receiver signal was achieved. High spikes were observed in the frequency response shortly after N_2_ was injected to desorb the sensor, which was caused by instantly increased air pressure in the chamber. Figure 6 shows the calibration curve of the nano-Ni(OH)_2_-coated QCM sensor to different DBP concentrations. The mass of DBP molecular adsorbed to the nano-Ni(OH)_2_ film increased linearly when the concentration was between 0.4 ppb and 16 ppb. Then, with the concentration increased, the increments of the mass adsorbed become fewer. The sensitivity was achieved as 2.4 Hz/ppb, which is the same as the previous work [14], showing that the wireless method of data acquisition would not affect the adsorption process itself. Finally, the limit of detection of the sensor was achieved as 1.2 ppb (calculated as three times the signal-to-noise ratio).

### 3.5. Experiment at 3rd Harmonics

**Figure 7:**
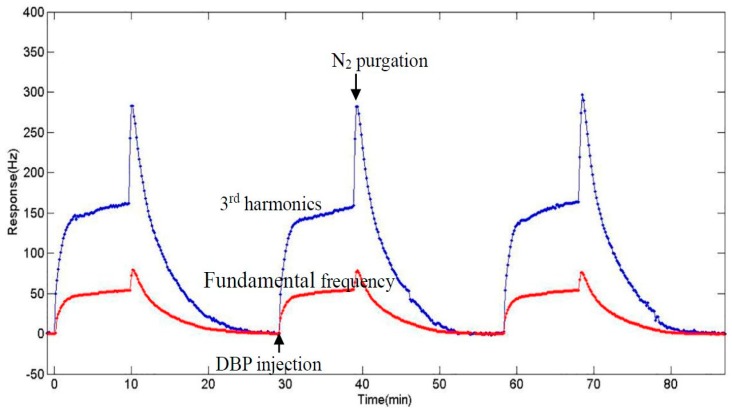
Responses of the nano-Ni(OH)_2_ QCM sensor to 24 ppb DBP. The red line is the response when the quartz oscillator works at the fundamental frequency, while the blue one works at 3rd harmonics.

**Figure 8:**
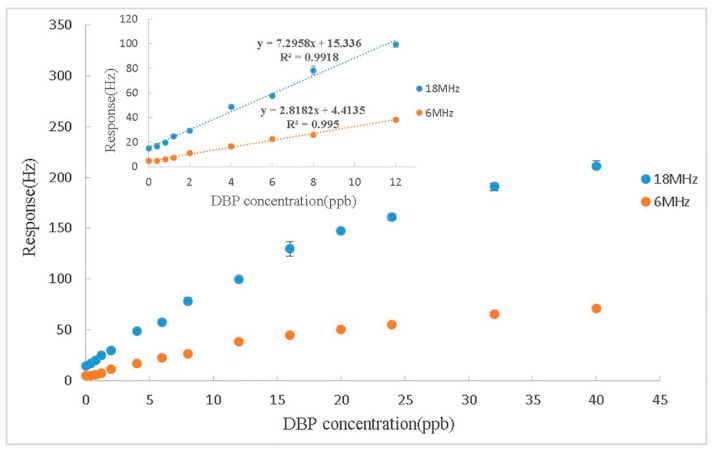
Calibration curve of the nano-Ni(OH)_2_-coated QCM sensor to different concentrations of DBP vapor. The red line is the response when the quartz oscillator works at the fundamental frequency, while the blue one works at 3rd harmonic. The inset indicates the calibration curve of the linear range.

With the noncontact configuration, operating at higher harmonics becomes a realistic way to improve the performance of the DBP sensor. We simply changed the frequency of the RF signal to about 18 MHz (the 3rd harmonic of the quartz plate), and carried out the same experiment. Figure 7 shows the responses of 24 ppb DBP when operating at the fundamental frequency and the 3rd harmonics, respectively. The frequency shifts were three times larger when the QCM sensor was operated at the 3rd harmonics than when being operated at the fundamental frequency, while the response time and the recovery time were exactly the same. This means that changing the operating frequency would not affect either the vapor adsorption process or mechanism, but only made the response of frequency increase three times and thus increased the sensitivity of the sensor. It is interesting that the height of the spike when operated at 3rd harmonics was far more than three times of its value when being operated at the fundamental frequency. This confirms that the spike in frequency when N_2_ started to inject to desorb the sensor was not caused by a mass increase, but by an air pressure change because of their different growth multiples. Therefore, we can speculate that different growth multiples in frequency shifts when operated in 3rd harmonics may help to research more in the kinetics of the absorption and desorption process of the vapor molecule and the sensing film, and more research needs to be done in the future. Figure 8 shows the calibration curves of the nano-Ni(OH)_2_-coated QCM sensors to DBP vapor when operated at different frequencies (the fundamental frequency and the 3rd harmonics). It implies that the sensitivity of the sensor being operated at the 3rd harmonics is three times of that when being operated at the fundamental frequency. The limit of detection working at the fundamental frequency the value was 1.2 ppb, while it was about 0.4 ppb when working at the 3rd harmonics (calculated as three times the signal-to-noise ratio). From all the data above and compared to the previous work, the use of the wireless-electrodeless QCM-D sensor with simply increasing the frequency of the RF signal could achieve better sensitivity and a lower limit of detection by using exactly the same sensing material, which means if better sensing film was not found yet, we could have an alternative to improve the sensing performance.

## 4. Conclusions

In this paper, a DBP gas sensor based on a homemade wireless-electrodeless QCM-D is present. With the noncontact configuration, wires or electrodes were removed so that damage to the chamber for wire was avoided and the operated frequency was raised to 3rd harmonics. Nano-structured nickel hydroxide [14] was used as the sensing film. With the 3rd harmonics operating frequency (18 MHz), a higher sensitivity of 7.3 Hz/ppb was achieved with half the usage of the sensing material. A low limit of detection 0.4 ppb was also achieved, which was below the safety concentration level and satisfied the detecting demand. Additionally, the system showed more stable than the traditional QCM system owing to the fitting process when calculating the frequency.

In the future, more work needs to be done to improve the performance of this new kind of wireless-electrodeless QCM sensor. Using a thinner quartz plate and operating in higher harmonics will be tried to achieve a better performance of the gas sensor. Moreover, the potential for obtaining more information about the experiment subject through simultaneously detecting the frequency and the dissipation value will be researched.
